# Emergence and transmission dynamics of the FY.4 Omicron variant in Kenya

**DOI:** 10.1093/ve/veaf035

**Published:** 2025-05-11

**Authors:** Sebastian Musundi, Mike Mwanga, Arnold Lambisia, John M Morobe, Nickson Murunga, Edidah Moraa, Leonard Ndwiga, Robinson Cheruiyot, Jennifer Musyoki, Martin Mutunga, Laura M Guzman-Rincon, Charles Sande, Joseph Mwangangi, Philip Bejon, Lynette Isabella Ochola-Oyier, David James Nokes, Charles N Agoti, Joyce Nyiro, George Githinji

**Affiliations:** Department of Epidemiology & Demography, KEMRI-Wellcome Trust Research Programme, P.O. Box 230-80108, Kilifi, Kenya; Department of Epidemiology & Demography, KEMRI-Wellcome Trust Research Programme, P.O. Box 230-80108, Kilifi, Kenya; Department of Epidemiology & Demography, KEMRI-Wellcome Trust Research Programme, P.O. Box 230-80108, Kilifi, Kenya; Department of Epidemiology & Demography, KEMRI-Wellcome Trust Research Programme, P.O. Box 230-80108, Kilifi, Kenya; Department of Epidemiology & Demography, KEMRI-Wellcome Trust Research Programme, P.O. Box 230-80108, Kilifi, Kenya; Department of Epidemiology & Demography, KEMRI-Wellcome Trust Research Programme, P.O. Box 230-80108, Kilifi, Kenya; Department of Epidemiology & Demography, KEMRI-Wellcome Trust Research Programme, P.O. Box 230-80108, Kilifi, Kenya; Department of Epidemiology & Demography, KEMRI-Wellcome Trust Research Programme, P.O. Box 230-80108, Kilifi, Kenya; Department of Epidemiology & Demography, KEMRI-Wellcome Trust Research Programme, P.O. Box 230-80108, Kilifi, Kenya; Department of Epidemiology & Demography, KEMRI-Wellcome Trust Research Programme, P.O. Box 230-80108, Kilifi, Kenya; Department of Epidemiology & Demography, KEMRI-Wellcome Trust Research Programme, P.O. Box 230-80108, Kilifi, Kenya; School of Life Sciences and Zeeman Institute for Systems Biology and Infectious Disease Epidemiology Research (SBIDER), University of Warwick, Coventry CV4 7AL, United Kingdom; Department of Epidemiology & Demography, KEMRI-Wellcome Trust Research Programme, P.O. Box 230-80108, Kilifi, Kenya; Nuffield Department of Medicine, University of Oxford, Old Road Campus Oxford, OX3 7BN, United Kingdom; Department of Epidemiology & Demography, KEMRI-Wellcome Trust Research Programme, P.O. Box 230-80108, Kilifi, Kenya; Department of Epidemiology & Demography, KEMRI-Wellcome Trust Research Programme, P.O. Box 230-80108, Kilifi, Kenya; Nuffield Department of Medicine, University of Oxford, Old Road Campus Oxford, OX3 7BN, United Kingdom; Department of Epidemiology & Demography, KEMRI-Wellcome Trust Research Programme, P.O. Box 230-80108, Kilifi, Kenya; Nuffield Department of Medicine, University of Oxford, Old Road Campus Oxford, OX3 7BN, United Kingdom; Department of Epidemiology & Demography, KEMRI-Wellcome Trust Research Programme, P.O. Box 230-80108, Kilifi, Kenya; School of Life Sciences and Zeeman Institute for Systems Biology and Infectious Disease Epidemiology Research (SBIDER), University of Warwick, Coventry CV4 7AL, United Kingdom; Department of Epidemiology & Demography, KEMRI-Wellcome Trust Research Programme, P.O. Box 230-80108, Kilifi, Kenya; School of Public Health and Human Sciences, Pwani University, P.O. Box 195-80108, Kilifi, Kenya; Department of Epidemiology & Demography, KEMRI-Wellcome Trust Research Programme, P.O. Box 230-80108, Kilifi, Kenya; Department of Epidemiology & Demography, KEMRI-Wellcome Trust Research Programme, P.O. Box 230-80108, Kilifi, Kenya; Department of Biochemistry and Biotechnology, Pwani University, P.O. Box 195-80108, Kilifi, Kenya

**Keywords:** FY.4, Omicron, Kenya, phylogenetics

## Abstract

The recombinant FY.4 severe acute respiratory syndrome coronavirus 2 (SARS-CoV-2) variant was first reported in Kenya in March 2023 and was the dominant circulating variant between April and July 2023. The variant was characterized by two important mutations: Y451H in the receptor-binding domain of the spike protein and P42L in open reading frame 3a. Using phylogenetics and phylodynamic approaches, we investigated the emergence and spread of FY.4 in Kenya and the rest of the world. Our findings suggest FY.4 circulated early in Kenya before its export to North America and Europe. Early circulation of FY.4 in Kenya was predominantly observed in the coastal part of the country, and the estimated time to the most recent common ancestor suggests FY.4 circulated as early as December 2022. The collected genomic and epidemiological data show that the FY.4 variant led to a large local outbreak in Kenya and resulted in localized outbreaks in Europe, North America, and Asia-Pacific. These findings underscore the importance of sustained genomic surveillance, especially in under-sampled regions, in deepening our understanding of the evolution and spread of SARS-CoV-2 variants.

## Introduction

Severe acute respiratory syndrome coronavirus 2 (SARS-CoV-2) emerged in Wuhan, China, in late 2019 (Lu et al. 2020, Zhu et al. 2020). Over the past 5 years, SARS-CoV-2 has evolved into multiple lineages, some more transmissible than others, reducing the effectiveness of public health interventions such as therapeutics, diagnostics, and vaccines (Centre for Disease Control and Prevention 2021). The World Health Organization (WHO) designated these lineages as variants of concern (VOC) (https://www.who.int/activities/tracking-SARS-CoV-2-variants). The VOC include Alpha (B.1.17) (Hill et al. 2022), Beta (B.1.35) (Tegally et al. 2021), Gamma (P.1) (Faria et al. 2021), Delta (B.1.617.2) (Cherian et al. 2021), and Omicron (Viana et al. 2022). The Omicron variant was first reported on 24 November 2021 in South Africa and Botswana and carried over 30 mutations in the spike protein compared to the Wuhan-Hu-1 strain (Tegally et al. 2022, Viana et al. 2022). These mutations increased transmissibility and reduced neutralization by anti-SARS-CoV-2 antibodies (Planas et al. 2022). While the origin of Omicron remains debatable, possible hypotheses explaining its emergence include extreme under-sampling resulting in undetected lineages, zoonotic spillover from animals that transmit SARS-CoV-2 without an intermediate host like the white-tailed deer and farmed mink, and persistence of the virus in chronically infected individuals (Markov et al. 2023).

The Omicron variant was initially classified into three lineages: BA.1, BA.2, and BA.3, before the emergence of BA.4 and BA.5 (Tegally et al. 2022). Unlike previous waves of VOCs, which were driven by a few circulating variants (Li et al. 2021), the emergence of Omicron led to convergent evolution and diversification (Andreis et al. 2023, Ito et al. 2023), resulting in multiple co-circulating lineages and an increased probability of recombination events. In mid-August 2022, the XBB recombinant was reported in India, Singapore, and other parts of Asia (Wang et al. 2023). XBB emerged from two BA.2 Omicron variants BM.1.1.1 (BA.2.75.3.1.1.1) and BJ.1 (BA.2.10.1.1) with breakpoints located in the receptor-binding domain (RBD) of spike protein (Tamura et al. 2023). XBB showed increased immune resistance, fusogenicity, and binding affinity to angiotensin-converting enzyme 2 (ACE2), the receptor mediating viral infection, compared to the parental strains (Tamura et al. 2023). Since then, multiple XBB variants, such as XBB.1.5 (Parums 2023) and XBB.1.16 (Looi 2023), have emerged and spread globally.

The FY.4 (XBB.1.22.1.4) Omicron variant became the dominant lineage in Kenya between March and July 2023 in Kenya following its initial detection on 10 March 2023 (Mwanga et al. 2023). Globally, the first FY.4 sample outside Kenya was reported on 3 April 2023 in Germany (Khare et al. 2021). In this work, we aimed to understand the origin and transmission of the FY.4 Omicron subvariant using genomic data. FY.4 circulated as early as December 2022 and was observed in Kenya first before being exported to other regions of the world. These findings suggest FY.4 potentially originated in Kenya and underscore the importance of genomic surveillance in tracking SARS-CoV-2 variants.

## Methods

### Data sources

We compiled a dataset of circulating SARS-CoV-2 sequences in Kenya between October 2022 and October 2023 (*n* = 1078). Out of these, 73 genomes classified as FY.4 were sequenced by the Kenya Medical Research Institute Wellcome Trust Research Programme (KWTRP) as previously described (Mwanga et al. 2023). An additional (*n* = 139) sequenced within Kenya and across the globe (*n* = 755) were retrieved from Global Initiative on Sharing All Influenza Data (GISAID) (*n* = 755). This global dataset is summarized in Supplementary Table 1. FY.4 lineages and XBB consensus genomes were retrieved from an online open resource (https://github.com/corneliusroemer/pango-sequences) and aligned using MAFFT. Amino acid mutations relative to the Wuhan-HU-1 and XBB were plotted using SNIPit (O’Toole et al. 2024).

### Time-scaled analysis of the FY.4 variant

Sequences with less than 70% genome coverage (*n* = 1), incomplete collection dates (*n* = 11), and whose lineage were misclassified based on Nextclade v.3.8.2 (*n* = 5) were excluded from the combined FY.4 datasets. The remaining sequences were aligned against the SARS-CoV-2 (XBB) reference (a Wuhan-Hu-1/2019 reference (Accession number MN908947.3) containing XBB single nucleotide polymorphisms) using MAFFT v.6.240 (Katoh and Standley 2013). The alignment was visualized using AliView v.1.28 (Larsson 2014), and the 5′ and 3′ ends containing the untranslated regions were manually trimmed. A maximum likelihood (ML) tree was generated using IQ-TREE v.2.3.3 with 1000 bootstrap values using the General Time Reversible (GTR) model (Nguyen et al. 2015). The ML tree was visualized using the R package ‘ggtree’ and low-quality sequences (*n* = 16) dropped from the tree using the R package ‘ape’.

### Phylogeography analysis

A time-resolved tree was generated using TreeTime v.0.11.4 (Sagulenko et al. 2018), and the presence of a molecular signal was evaluated using TempEst v.1.5.3 (Rambaut et al. 2016) followed by a Bayesian analysis with BEAST (Drummond and Rambaut 2007). To identify the clades circulating within Kenya, a discrete phylogeography approach utilizing two locations, ‘Kenya’ and ‘others’, was conducted in BEAST 1.10.4. This approach used the time-resolved phylogeny as the starting tree to reduce the computational time needed to generate transmission information (Bollen et al. 2021). Bayesian inference was run with BEAST v1.10.4 for 6 × 10^5^ Monte Carlo Markov Chain (MCMC) steps, sampling every 1000 steps. Log files were examined for convergence and mixing using Tracer v1.7.1 (Rambaut et al. 2018). After discarding 10% as burn-in, a maximum clade credibility (MCC) tree was generated using TreeAnnotator v.1.10.4 (Suchard et al. 2018). The overall number of transitions between the two locations was counted in the BEAST package Babel using sampled trees. Since Kenyan samples were detected earlier than global sequences, we repeated the above analysis with samples collected from May 2023 and counted the transitions between ‘Kenya’ and ‘others’.

To explore the local transmission of the FY.4 variant within Kenya, a relaxed random walk (RRW) diffusion model, which allows for dispersal velocity in the tree to vary but remain the same in the branches, was inferred using the longitude and latitudes of sample locations as continuous traits (Lemey et al. 2010, Pybus et al. 2012). Since the RRW model does not allow samples to have the same geographical coordinates, we selected the centroid location of each administrative point and added a random jitter of 0.05 to each tip, making each location distinct. An MCMC chain was run in duplicate for 500 million iterations sampled after every 50 000 steps, with its mixing properties checked in Tracer v.1.7.1 to ensure the effective sample size (ESS) was >200. The R package ‘seraphim’ was used to extract spatiotemporal information embedded in the posterior MCC trees and visualizes the dispersal of FY.4 within Kenyan counties (Dellicour et al. 2016).

Next, we carried out a discrete phylogeographic analysis to infer the global origin and movement of FY.4 across different regions. We aggregated the country of sampling into four regions, including Kenya (*n* = 206), North America (*n* = 494), Europe (*n* = 140), and Asia-Pacific (Asia and Oceania) (*n* = 91). One sample from South America and two from Uganda were excluded to minimize biased posterior distributions due to unequal sample size. We analysed samples collected from March 2023, aiming to capture the spread of FY.4 from the time it was initially identified, thereby providing a clear evolutionary trajectory of the geographic spread of FY.4 while in circulation. Since a large number of sequences originated from the USA, down sampling was carried out based on time, Pango lineage, and region, resulting in a total of 511 sequences distributed as follows: Kenya (*n* = 94), North America (*n* = 233), Europe (*n* = 107), and Asia-Pacific (*n* = 77). Ancestral location reconstruction of the FY.4 variant and asymmetric viral exchanges between regions were estimated utilizing a non-continuous time Markov chain model. Using a Bayes factor test, the Bayesian stochastic search variable selection was used to infer non-zero migration rates and identify the best-supported transition rates. In addition, Markov jump counting was used to estimate the number of transitions across the different regions. The time-scaled phylogeny was created using the HKY substitution model with a gamma-distributed rate for invariant sites, an uncorrelated relaxed molecular clock with a log-normal prior, and a Bayesian sky grid with 52 change points over 1 year. The analysis was run in duplicate on BEAST v1.10.4 using 200 million MCMC steps with sampling after every 20 000 steps. Mixing and convergence properties were assessed using Tracer v.1.71, ensuring the ESS was >200. After discarding the 10% burn-in, the MCC tree was constructed using TreeAnnotator v.1.10, and the number of transitions/Markov jumps was estimated using TreeMarkovJumpHistoryAnalyzer (Lemey et al. 2021).

## Results

### FY.4 circulated in Kenya before global expansion and underwent rapid evolution over a short period

**Figure 1:**
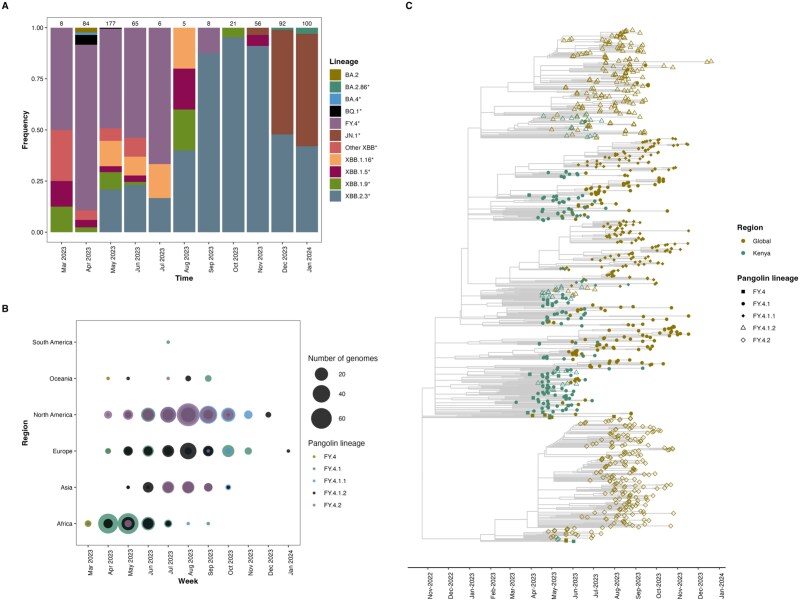
Circulation of the FY.4 Omicron subvariant. (A) Proportion of SARS-CoV-2 lineages circulating in Kenya from March 2023 to January 2024, the total number of sequences deposited each month in GISAID is shown on top of the stacked bar plot. (B) Global distribution of circulating FY.4 variants between March 2023 and January 2024, the individual colours represent the FY.4 sub-lineages while the circle size denotes the number of genomes deposited in GISAID each month. (C) Time-resolved phylogeny showing the temporal clustering of FY.4 sequences from Kenya and the rest of the world, the colours represent the region from which the sample was collected, while the shape indicates the FY.4 Pango sub-lineages.

The FY.4 variant became the dominant circulating lineage in Kenya between March and July 2023 before its eventual replacement with other Omicron subvariants (Fig. 1A). This period coincides with the high positivity rate observed from samples collected from health facilities within the Kilifi Health Demographic Surveillance System (KHDSS) (Supplementary Fig. 1). On the global scale, FY.4 was first detected in Africa before its eventual detection in other continents across the globe (Fig. 1B). In Africa, FY.4 circulation peaked between April and May 2023; in North America and Europe, the peak was observed in July 2023 (Fig. 1B). In Africa, sub-lineage FY.4.1 was the dominant circulating lineage and co-circulated with its expanded lineage FY.4.1.2. North America initially saw FY.4.1 and FY.4.2 in April 2023, followed by FY.4.1.2 before the co-circulation of FY.4.2 and FY.4.1.1 in the later stages. FY.4.1 and FY.4.1.2 were the main circulating lineages in Europe from April 2023. While the same lineages were initially detected in Asia from May 2023, FY.4.2 became the main circulating lineage thereafter from June to September 2023 (Fig. 1B).

Phylogenetic analysis of the circulating strains showed that FY.4 was closely related to other XBB variants, specifically XBB.1.16 and XBB.1.9 (Supplementary Fig. 2). As expected, FY.4 was also present in the same clade as BA.2 and BA.2.75, which had previously been proposed as the main parental lineages for the emergence of the recombinant XBB. In addition to the two lineage-defining mutations—Y451H in the spike protein and P42L in ORF3a—expanded sub-lineages exhibited notable mutations. FY.4.1 contained S494P in the RBD of the spike glycoprotein while FY.4.1.1 included S704L in subdomain 2. The FY.4.1.2 sub-lineage was characterized by S2926F in ORF1a, and FY.4.2 displayed mutations Y2171F in ORF1a and V2287I in ORF1b.

A time-resolved phylogeny containing Kenyan (*n* = 207) FY.4 and global sequences (*n* = 738) revealed a short period between the expansion of FY.4.1 to FY.4.1.1 and FY.4.1.2, suggesting a rapid diversification process (Fig. 1C). Following its expansion, FY.4.1 persisted for 36 weeks in circulation with its derived lineages. In contrast, FY.4.2, which also emerged from FY.4, did not expand to additional lineages, likely reflecting the different selective pressures acting on individual FY.4 lineages. Analysis of the temporal spread revealed that FY.4 circulated early in Kenya compared to other regions of the globe (Fig. 1C). Additionally, Kenyan FY.4 sequences displayed significant genetic diversity by mapping to multiple lineages and formed clusters, providing evidence for local transmission ahead of introductions to other regions (Fig. 1C).

### The FY.4 variant originated in Kenya

**Figure 2:**
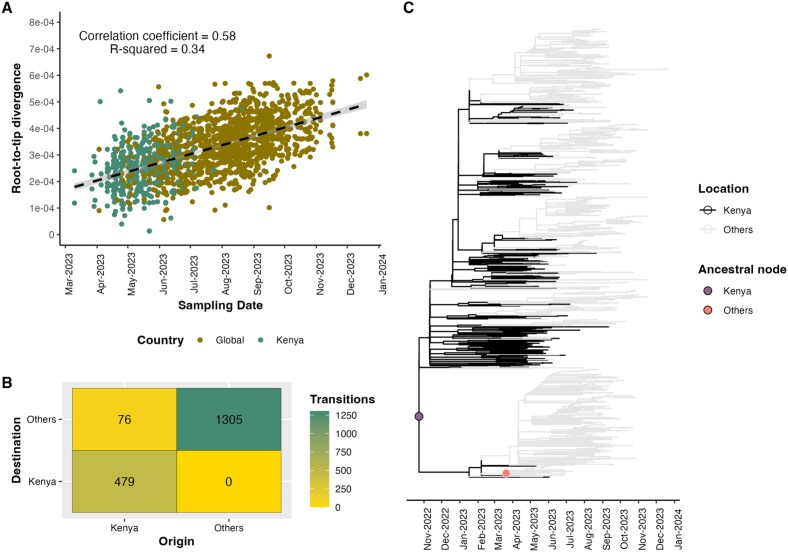
(A) Root-to-tip regression plot generated from a TempEst analysis, showing evidence of a temporal signal (*R*^2^ = 0.326, correlation coefficient = 0.57), the FY.4 sequences are coloured according to the locations (Kenya or Global) and the regression line represents the estimated mean evolutionary rate with error buffers in grey, showing the 90% confidence intervals. (B) The number of transitions was derived from the MCC tree by counting changes between ‘Kenyan’ and ‘other’ locations, transitions were counted when the location of the internal node changed from ‘Kenya’ to ‘Others’ or *vice versa* or when maintained in the same position. (C) Preliminary discrete trait analysis identified two ancestral clades associated with the spread of FY.4 using the time-resolved tree as the starting tree, the black colour represents the most probable location for Kenyan sequences and background grey the most probable location for global sequences, two ancestral nodes are filled by the plum and salmon colours respectively and the ‘other’ ancestral node occurs primarily on the FY.4.2 sub-lineage, which was not largely observed in Kenya but was predominant in North America.

The presence of a temporal signal was confirmed by the linear relationship between genetic divergence and sampling dates using TempEst v.1.5.3 (*R*^2^ = 0.34) (Fig. 2A). Subsequently, we carried out a discrete phylogeographic analysis to identify the potential origin and the number of import and export events to Kenya using the FY.4 variant using genomic data. Based on this, we observed no evidence of independent introductions of FY.4 in Kenya (Fig. 2B) based on the 95% highest posterior density (HPD). However, we identified at least 75 transmission events from Kenya to the rest of the globe (95% HPD interval = [72–79]). Local transmission dynamics in Kenya comprised over 479 transition events between counties (95% HPD interval = [475–484]) (Fig. 2B). Evidence of spread is supported by export and imports events between the counties. Global spread was observed with support for multiple export events {1304 transition events (95% HPD interval = [1296–1311]) across the globe showing FY.4 arrived and transmitted across multiple regions}. As shown in Fig. 1B, many early sequences were sampled from Kenya and could influence the emergence and spread of FY.4, as depicted in Fig. 2B. To better assess the impact of these early sequences, we repeated the phylogeographic analysis using data sampled from May 2023 onwards. The results supported the previous analysis, showing no inferred introductions to Kenya while an average of 68 export events were reported from Kenya to other regions (95% HPD interval = [66–72]) (Supplementary Fig. 3).

Ancestral reconstruction analysis revealed the presence of two most common recent ancestors. The first node infers Kenyan ancestry and includes all Kenyan sequences alongside global sequences, forming the largest clade with 915 sequences (Fig. 2C). The genetic diversity of the Kenyan sequences in this node supports strong local transmission within Kenya, while the presence of both Kenyan and global sequences in the same node suggests possible worldwide dissemination of the FY.4 variant. In contrast, the second ancestral node was associated with fewer sequences (*n* = 19) and likely represented a more localized transmission route. Notably, the second ancestral node did not include Kenyan samples, which might suggest limited or no transmission to or from Kenya, or a pathway that completely bypassed Kenya (Fig. 2C).

**Figure 3:**
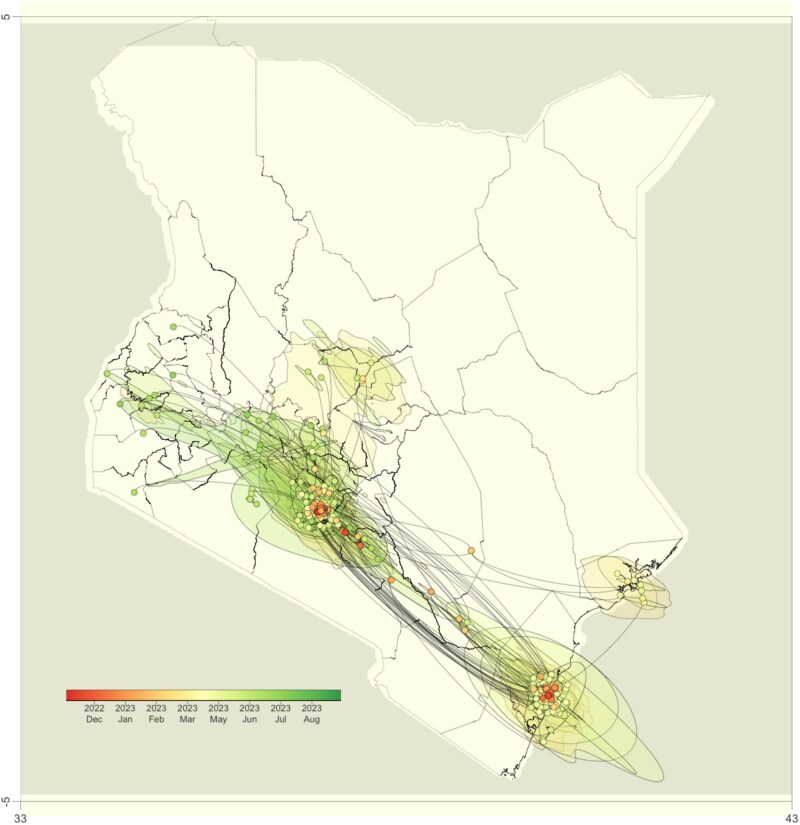
Dispersal of FY.4 across Kenya over time based on 1000 subsampled trees from a continuous phylogeographic posterior distribution, the nodes of the MCC tree are colour-coded based on the time of occurrence, and the 80% HPD regions are displayed in successive layers with the colours reflecting corresponding time periods for virus spread.

We extracted the ancestral clade that inferred Kenyan ancestry and examined virus dispersal within Kenya. The subsequent continuous phylogeography of dispersal patterns within Kenya supported the evidence that FY.4 was the dominant circulating lineage in the coastal region from March 2023. By May–June 2023, most cases were observed centrally in the capital city (Fig. 3). Most peripheral samples also directly connected to either the coastal region or centrally in the capital city, which were the predominant sampling sites. This pattern likely suggests a limited geographical range of sampling in Kenya, potentially leading to an underestimate of the circulation of FY.4 in other parts of Kenya.

**Figure 4:**
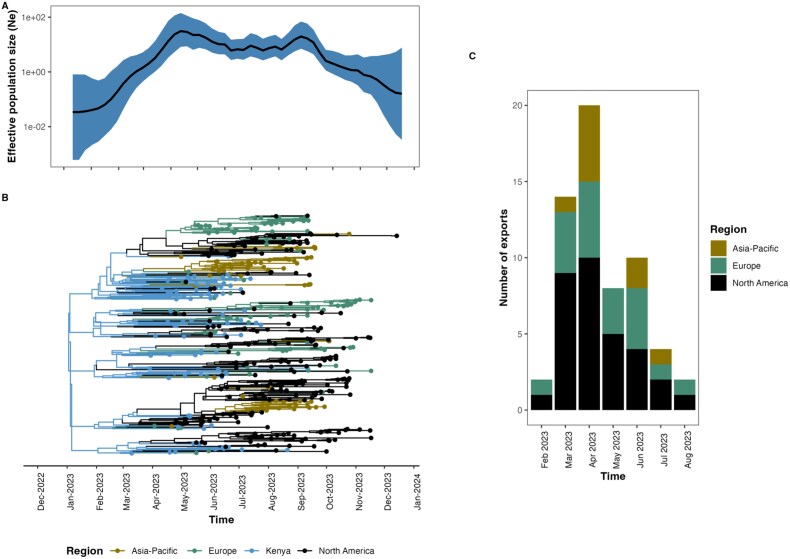
Bayesian phylogeographic reconstruction of FY.4. (A) A Bayesian Skyline plot describing the inferred change in the effective population size of FY.4 infections over time. (B) A time-resolved maximum credibility clade tree with branches coloured by inferred geographic location. (C) A summary of the number of Markov Jumps observed from Kenya to other regions stratified by months.

Bayesian skyline analysis revealed an exponential increase in the effective population size between January and April 2023 followed by a minor decline and another slight exponential increase from August to September before a decline to January 2024 (Fig. 4A). Phylogeographic reconstruction analysis implied that FY.4 may have emerged in Kenya in early January (mean tMRCA 3 January, 95% HPD 2 December 2022–1 February 2023) across the two replicates (Fig. 4B). We estimated that FY.4 was exported out of Kenya on at least 60 occasions with majority of the events to North America (*n* = 32), followed by Europe (*n* = 19) and lastly Asia-Pacific (*n* = 9) (Fig. 4C). Based on our analysis, FY.4 was first exported to Europe and North America in February 2023, followed by an increase in these two regions and an introduction in Asia-Pacific in March 2023. The number of export events (*n* = 20) peaked in April 2023 (Fig. 4C). There were no inferred introductions from the globe to Kenya during this period.

## Discussion

The FY.4 variant increased in circulation in Kenya between March and July 2023 accounting for increased cases and hospitalization (Mwanga et al. 2023). This variant was also observed in other regions across the globe (Fig. 1C). Given the early isolation of FY.4 and increased transmission intensity in Kenya, we applied genomic and epidemiological data to investigate the emergence and transmission dynamics of FY.4 variant from sequenced samples in Kenya in addition to those collected from the globe.

The FY.4 lineage of SARS-CoV-2 is characterized by two notable mutations: namely, the Y451H in the RBD of the spike protein, whose functional implication is unclear, and the P42L in the ORF3a, with potential for contribution to the loss of recognition of T-cell epitopes (de Silva et al. 2021). Additionally, the S494P present in the RBD of FY.4.1 enhances binding affinity to ACE2 receptor, increasing transmissibility (Chakraborty 2021) and potentially contributing to the increased number of observed cases. Neutralization assays against multiple circulating Omicron variants, including FY.4 in Kenya between March 2023 and March 2024, have provided evidence for a decline in naturally acquired and vaccine-mediated antibody responses, implying that the Kenyan population was still susceptible to infections caused by emerging Omicron subvariants (Lugano et al. 2024). Here, we used an established health facilities surveillance platform (Nyiro et al. 2018), and combined this with further local and global genomic data to determine the origin and describe the transmission dynamics of this variant.

The phylogenetic and phylogeographic reconstruction suggests that Kenya was the potential origin of the FY.4 variant (Fig. 2B). Phylogenetic and phylogenomic analysis provides evidence for multiple exportation events from Kenya, primarily to North America and Europe (Fig. 3B) between March and July 2023. Previously, we have observed multiple introductions of ancestral strains (Githinji et al. 2021) and VOC (Agoti et al. 2022, Githinji et al., 2025, unpublished data). The Bayesian skyline plot showed that the effective population size corresponded with an increase in the number of cases and potentially indicated missed cases during the outbreak period. A peak in the effective population size was observed between August and September 2023, suggesting a surge in cases outside of Kenya coinciding with a rise in the FY.4 genome sequences deposited from North America and Europe (Fig. 3C). This suggests ongoing transmission events between Kenya and the rest of the world following the initial emergence of FY.4.

A limitation with this study is that the true number of FY.4 derived COVID-19 cases in Kenya, Africa, and South America is likely underestimated given the limited number of tests at the period under-surveillance and the limited genomic surveillance in the country. The biased sampling is underscored by clustered outbreaks in areas with higher sampling rates.

## Conclusion

Genomic surveillance is critical in identifying emerging variants of SARS-CoV-2. In this study, the use of phylogenetics and phylodynamic approaches provided insights into the potential origin and dispersal patterns of the FY.4 SARS-CoV-2 variant. The study emphasizes the need for increased SARS-CoV-2 genomic surveillance and capacity in under-sampled geographies.

## Supplementary Material

Supplementary_file_clean_copy_veaf035

## Data Availability

The consensus genome sequences obtained in this study were submitted to both GISAID and GenBank databases and the accession numbers available in the supplementary material. The raw data files have been prepared for deposition in Harvard DataVerse (https://doi.org/10.7910/DVN/VPWUXN). For more detailed information beyond the metadata used in the paper, there is a process of managed access requiring submission of a request form for consideration by our Data Governance Committee.
