# Supplementary material for: Emergence and transmission dynamics of the FY.4 Omicron variant in Kenya
Source: Virus Evol. 2025 May 11;11(1):veaf035. doi: 10.1093/ve/veaf035 (PMC12202047; doi:10.1093/ve/veaf035)
Supplement: Supplementary_file_clean_copy_veaf035 [file supplementary_file_clean_copy_veaf035.docx]

**Supplementary files**


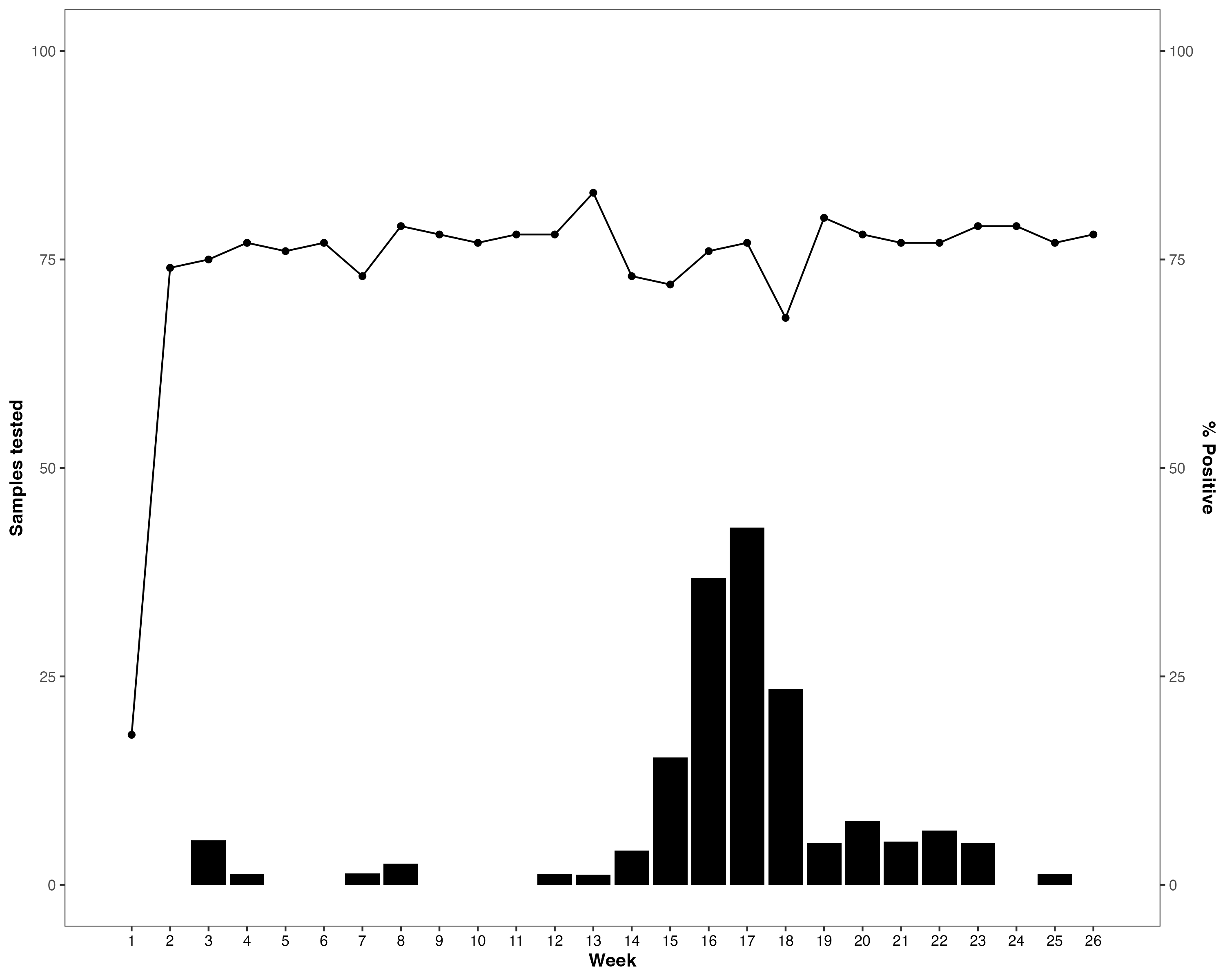


**Supplementary Figure 1.** The number of samples collected and tested and the positivity rate in KHDSS in the first 26 weeks of 2023. The line graph represents the number of samples collected weekly, while the bar graph shows the positivity rate within the same period. A total of 125 out of 1934 (0.06%) SARS-CoV-2 samples collected within KHDSS between January and July were positive for SARS-CoV-2, with the positivity rate peaking in April.


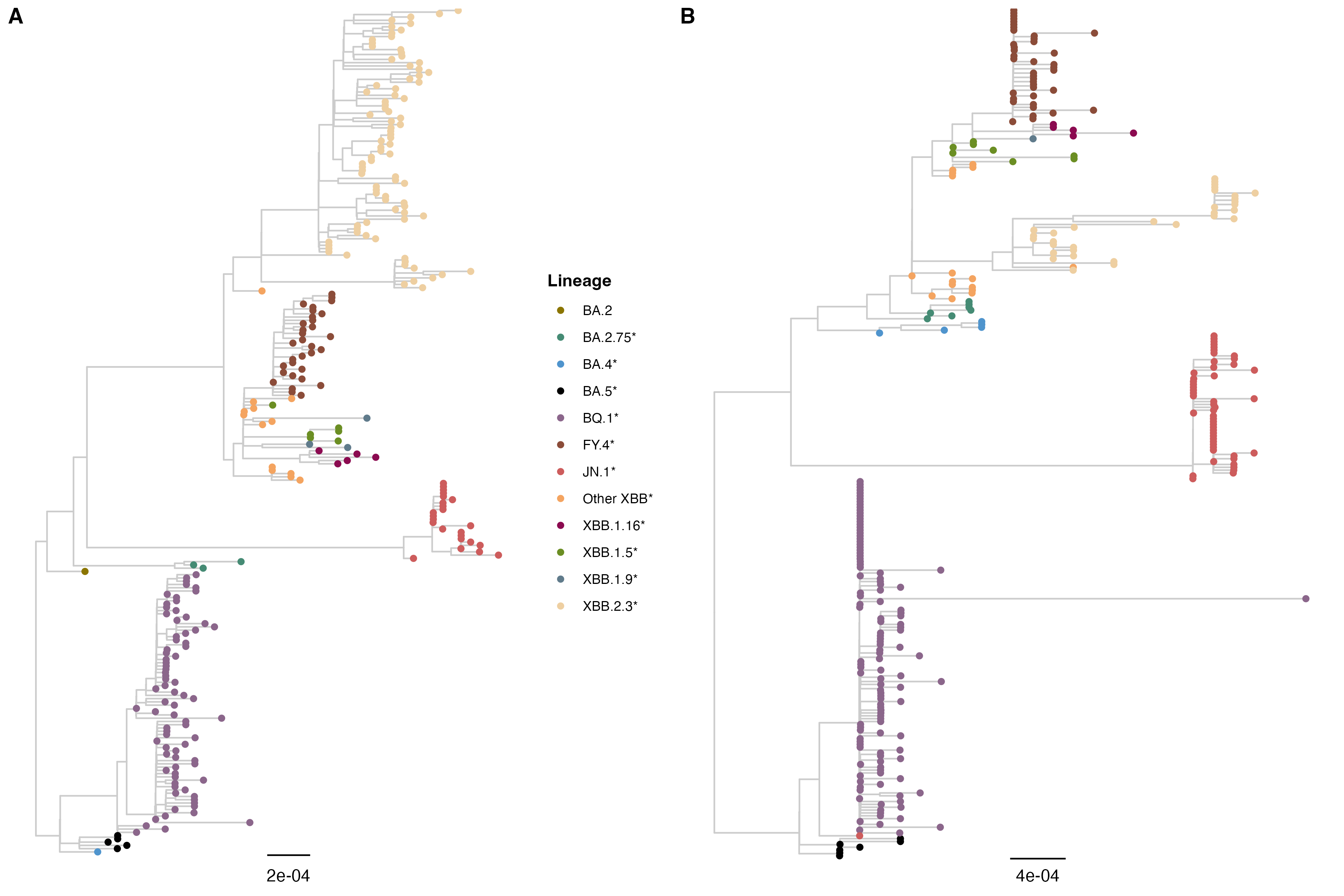


**Supplementary Figure 2.** Maximum likelihood (ML) phylogenies of SARS-CoV-2 sequences from Kenya, partitioned by the XBB recombination breakpoint at position 22,920. (A) ML tree inferred from the 5′ genomic region (positions 1–22,920). (B) ML tree inferred from the 3′ genomic region (positions 22,920–29,903). Tips are colour-coded according to Pango lineage classifications.

**
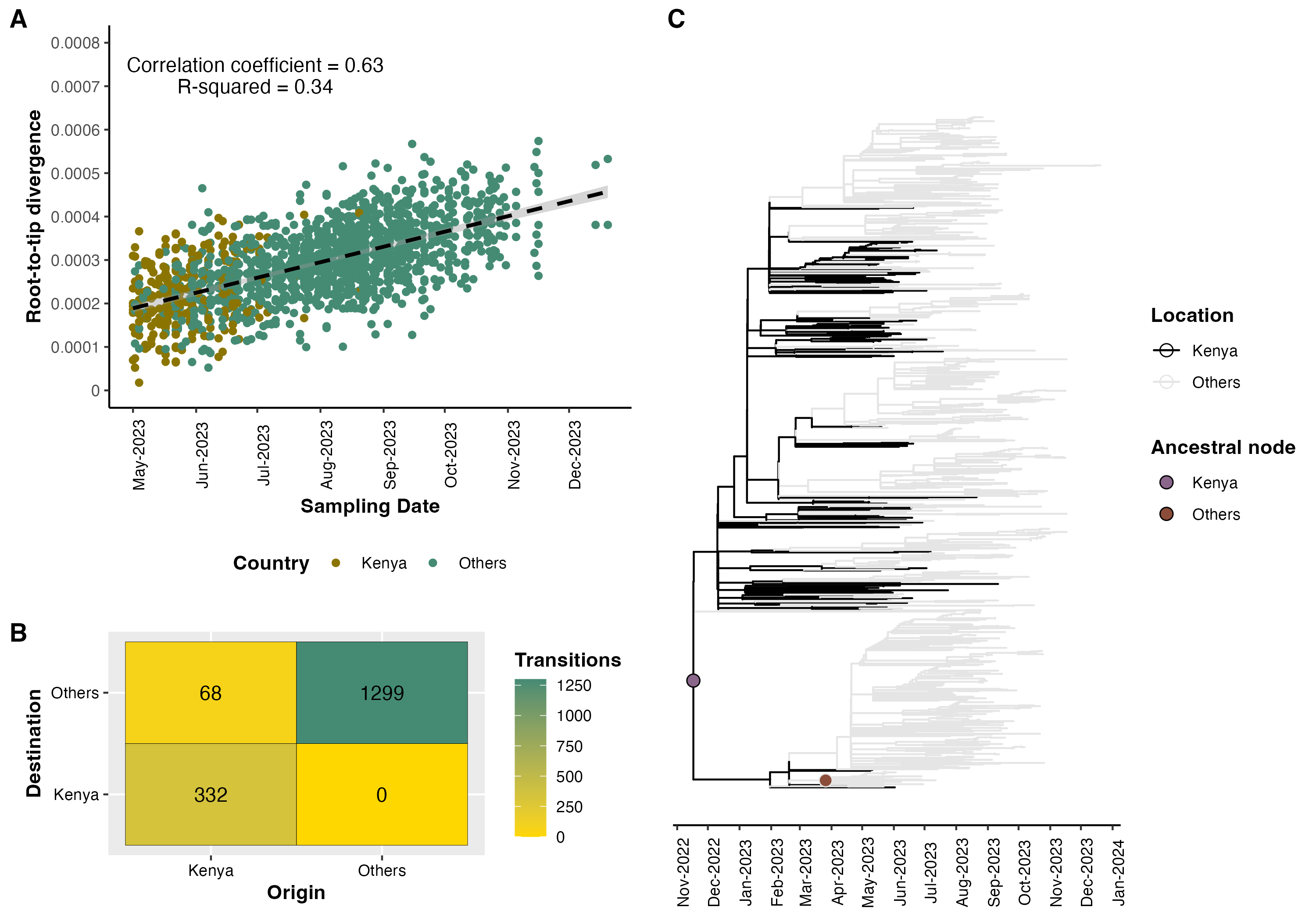
**

**Supplementary Figure 3.** Preliminary Bayesian phylogeographic analysis comparing transitions between Kenya and other regions from May 2023. A) Root-to-tip regression comparing a positive correlation between sampling dates and genetic divergence for sequences collected between May 2023 to January 2024. B) Number of transitions between Kenya and other locations from the preliminary discrete trait analysis. No inferred introductions are reported in Kenya, while an average of 68 export events are reported from Kenya to other regions (95% HPD interval = [66-72]). C) Kenyan ancestral node associated with multiple samples that appeared early during FY.4 circulation and suggests local establishment and onward transmission to other regions. The “Other” ancestral node was associated with fewer samples than the Kenyan ancestral node, possibly indicating a less significant role in disseminating FY.4.

**Supplementary Table 1**. The number of FY.4 sequences retrieved from GISAID stratified by the country of origin.

| **Country** | **Number of samples (n=755)** |
| --- | --- |
| Australia | 8 |
| Austria | 3 |
| Belgium | 1 |
| Brazil | 1 |
| Canada | 43 |
| China | 5 |
| Croatia | 3 |
| Denmark | 2 |
| France | 6 |
| Germany | 4 |
| Greece | 1 |
| India | 1 |
| Indonesia | 1 |
| Ireland | 10 |
| Israel | 2 |
| Italy | 3 |
| Japan | 39 |
| Netherlands | 1 |
| Portugal | 1 |
| Puerto Rico | 1 |
| Romania | 3 |
| Saudi Arabia | 1 |
| Slovenia | 3 |
| South Korea | 36 |
| Spain | 2 |
| Sweden | 19 |
| Switzerland | 3 |
| Uganda | 2 |
| United Kingdom | 80 |
| US | 469 |
